# The Vaccination Testimony

**Published:** 1897-06

**Authors:** M. R. Leverson

**Affiliations:** Fort Hamilton, N. Y.


### THE VACCINATION TESTIMONY.

M. R. LEVERSON, M. D., FORT HAMILTON, N. Y.

In connection with the abstract of the testimony taken before the Royal (Britsh) Commission on vaccination, now publishing in this journal, I desire to furnish Dr. James with copies of several of the diagrams supplied to the Commission by many of the witnesses who testified before them, as such diagrams present in an impressive manner the lessons to be learned from tables of great importance, while the tables themselves would be apt to tire the attention of the reader. Copies of two diagrams supplied to the Commission by Dr. Grimshaw, one of the most authoritative witnesses who testified in support of vaccination, have already been handed to Dr. James, and furnish, almost at a glance, the data on which that gentleman founded his opinions and by which those opinions are seen to be unfounded.

But to engrave and print in colors these and further diagrams will be a costly work, though once produced many of the plates could be utilized for my work on *The History and Pathology of Vaccination*, for the publication whereof subscriptions are now being sought. Any person sending to Dr. James the sum of $10.00 as a contribution toward the cost of engraving and printing these plates shall be credited as entitled to one copy in boards of the above-mentioned work. In the much to be desired (but unlikely) event of a larger amount being thus subscribed than needed to pay the cost of engraving and printing the diagrams, Dr. James has agreed to apply such surplus as subscriptions for the work, whose speedy publication will thus be rendered more probable.

Dr. James has also consented to receive the names of those who are willing to subscribe for my work, or they can communicate directly with me.

The MS., diagrams and drawings are ready for printer and engraver. The heavy expense of numerous colored illustrations renders it essential that enough subscriptions shall be had to guarantee against loss any publisher before he can be expected to undertake the publication.

